# Discharged. Not Where to Lay His Head

**Published:** 1886-10-02

**Authors:** George Fleming


### Discharged.

"NOT WHERE TO LAY HIS HEAD."

By George Fleming.

I.

"Here you are, Davidson. All right, guard. Third class; but it doesn't matter, and, by Jove! I wouldn't have missed this train for something!"

The workman on the opposite seat looked up sympathetically. "'Twas a narrow shave, that, sir. I was watchin' of you standin' there a talkin' to the other gentlemen, an' I was afraid you wouldn't do it. Beg your pardon, sir, but if that bundle's in your way I'll move it."

The grey-haired man addressed as Davidson had drawn a paper-covered volume from his pocket and was already engrossed in reading. He did not even look up; but his companion, who was younger, answered for him. "Oh, that's all right; let it alone. It does no harm there." He gave one quick, comprehensive glance at his neighbour's worn black coat, buttoned tightly over the meagre chest and narrow shoulders, at his very bright eyes and thin, eager face. "Are you going up to the West End for a job? You don't seem very fit," he said good-naturedly.

The workman (his name was Arthur Lemon, a carver and gilder of frames by trade) brightened up at being spoken to, with something of the instinctive satisfaction of a friendly dog. He readjusted the position of his hands and feet, and a smile,—a smile which expressed all the *naïve* confidence and joy of convalescence,—transfigured his haggard face. "Seven months and a fortnight of hospital, sir," his voice, which was husky when he began speaking, soon grew clear; "seven months and a fortnight, Bed 71, Ward B, —— Infirmary, Rotherhithe, that's been about my ticket."

"—— Infirmary, eh? Why, that's the workhouse, isn't it?"

"Well, sir—"

His face clouded over for a moment like a child's, and he rubbed his hands together nervously across his knee. "It *is* the workhouse, an' that's an undoubted fact, an' as such ain't to be contradicted nohow. But bless you! you couldn't have told the difference. Wards—why, it would just do anybody good to see how they keep 'em; and nurses and doctors too. He's a very clever gentleman, our doctor, Dr. White. He just saved my life, he did. Why, when they carried me in there, that's seven months and a fortnight to-day, the 30th of March it were, and snowing, I remember,—well, sir, if you'll believe me, I did not understand what they was doing to me no more than a baby; an' if you'll believe me, they didn't expect me to live through the night. The nurse has told me that often. 'Ah, Lemon,' says she, 'it's you that are the lucky one,' says she, 'for as sure as you're lying there, when they brought you in I never expected to hear you say thank 'ee.' Irish, she was, too; but the doctors think a lot of her. She's a very clever woman."

"What was the matter with you? Lungs gone wrong, eh?"

"Wrong, sir? Why, so to speak, they was gone entirely. Ah, I've taken a deal of nursing."

The young man threw away his cigarette, and glanced out of the window at the last new theatrical advertisement on the grimy station wall. There are no posters on the walls when you get past Shadwell; there is no one there whom it would be to anybody's profit to amuse. "You'll have to be careful of yourself for a bit," he remarked, yawning.

"That's just what the doctor was a sayin', sir. I went into his office this morning, just to say good-bye and sort of thank him, an' he was tellin' me what I was to do if the hæmorrhage come on agen—'Just you keep clear of drink, Lemon,' says he, 'an' you'll do. An' if you can't or you won't keep clear of it,' he says, 'why, you're a dead man before November.' But I ain't going in for anything more of that kind now. I'm a sober man, I am, except for a glass now and then, friendly and reasonable. But I am going to cut it altogether. I've signed the pledge, an' I've got it in my pocket, an' I told him so. Look here, sir. May be— If you've never seen it—"

He drew a printed card out of the lining of his pocket-book. "Perhaps you might wish to have a look at it, as a curiosity like," he said, showing all his white teeth in a conciliatory smile.

The young man put out his hand.

"So that's the famous pledge, is it? And a very good thing too," he said easily, turning it about and looking at the printing on the card.

"Ay, sir. That's the real thing; an undoubted fact, which as such ain't to be contradicted. I mean to frame it when I get a bit of time; just a neat gold beading and a glass, and hang it up in my room as an ornament."

He broke into a sharp paroxysm of coughing as he spoke, which left him with a spot of red on either cheek. "'Twas a lady who came to our ward gave me that to sign," he said, as soon as he could go on speaking; "she was very kind to me, and she gave me her address in Kensington. I'm going there now. For she said she'd give me a job of work straight off to begin on, till I've time to take a look around me and get on again at the old shop. Perhaps you may know of it, sir,—Tillotson's, in Oxford Street? I know the foreman very well; I've done a lot of work for him before now. They 'ad one job they put me on for two years—gilding all the drawing-room chairs and couches and picture frames and wall panels for one o' the Rothschilds—Louis Quinze fashion. There were a lot of us on it, and we were more than two years to finish that job."

The train stopped as he was speaking, and he turned to look out of the window. "Next station's mine. Well, sir, if you'll believe me, it does seem curious to feel oneself walking about again in the sunshine. Miss Thornhill—that's the lady I was telling you of—she'll be surprised to see me, too. Some of them gets out of hospital in the rain. That's rather hard on a man if he's been in his ward a long time, and hasn't got much of a place to go home to, and is weak like, may be, through lying in bed. Why, when I came out myself, you'd not think it, sir, but it took me all of 'arf an hour to walk down to the station.—But I don't know that I've ever seen a finer day than this, for the time o' year."

"You'll have to take care of yourself for a bit," the young fellow said again.

"I'll look after that, sir. I'm a goin' back again there," he pointed vaguely with his thumb over his shoulder; "I'm a goin' back next Tuesday, that's Visitors' Day, to see a mate o' mine, and Lord bless you, they won't know me!"

The train glided up to the platform of South Kensington Station. He clutched his bundle and got out rather hastily at the last moment. The sudden change of position brought the colour back to his face.

"He's got enough to say for himself, at any rate. And they write about the Great Inarticulate East End!" the young man observed, locking after him with a laugh.

He lit another cigarette. "I say, Davidson."

Mr. Davidson dropped his book upon his knee.

"Did you notice that fellow who just got out? Did you look at his face? And do you remember Hastings, old Jim Hastings of Christ Church? It's the most amazing likeness I ever saw. I felt all the time as if I were talking to old Hastings, down on his luck, and turned journeyman gilder."

"I remember the man you mean," Mr. Davidson answered, diliberately. "I never had much to do with him—a facile, talkative sort of fellow, who was always going to set the Thames on fire the week after next."

"Oh! come now. He wasn't over-steady, I'll admit. But such a pleasant, attractive sort of chap! musical and artistic, and all that. And he had lots of friends; any quantity of them. But such a likeness I never saw. Bar the workhouse and the gilding business, and I believe you could not have told him apart from this fellow. It's the same material developed East and West." He laughed. "I should like to see them together. I should like to see Hastings's face; Hastings thinks something of his own looks, I can tell you!"

Mr. Davidson picked up his book again.

"The same material, eh? Well," he said, "there is the whole of civilisation to protect the weakness of one of them. But how about the other one?"

*(To be continued.)*

